# Properties of Paperboard Coated with Natural Polymers and Polymer Blends: Effect of the Number of Coating Layers

**DOI:** 10.3390/foods12142745

**Published:** 2023-07-19

**Authors:** Thaís de Cássia Naitzel, Vitor Augusto dos Santos Garcia, Carla Alves Monaco Lourenço, Fernanda Maria Vanin, Cristiana Maria Pedroso Yoshida, Rosemary Aparecida de Carvalho

**Affiliations:** 1 Faculty of Animal Science and Food Engineering, USP—University of São Paulo, Street Duque de Caxias Norte 225, Pirassununga 13635-900, Brazil; thais.naitzel@usp.br (T.d.C.N.); carla.monaco@usp.br (C.A.M.L.); fernanda.vanin@usp.br (F.M.V.); 2 Faculty of Agricultural Sciences, UNESP—São Paulo State University, Street José Barbosa de Barros 1780, Botucatu 19082-080, Brazil; vitor.as.garcia@unesp.br; 3 Institute of Environmental, Chemical and Pharmaceutical Sciences, UNIFESP—Federal University of São Paulo, Rua São Nicolau 210, Diadema 09913-030, Brazil; cristiana.yoshida@unifesp.br

**Keywords:** paperboard, agar, chitosan, barrier properties, grease resistance

## Abstract

Paper is one of the packaging materials that presents a biodegradable character, being used in several areas; however, its barrier properties (gases and fat) and mechanics are reduced, which limits its application. Coating papers with synthetic polymers improve these properties, reducing their biodegradability and recyclability. The objective of this work was to develop and characterize coated paperboard, using the tape casting technique, with different ratios of film form agar–agar/chitosan (AA:CHI, 100:0, 50:50, and 0:100) and different numbers of coating layers (operating times for application of 14.25 min and 28.5 min for one and two layers, respectively). A significant reduction in water absorption capacity was found by applying a 0:100 coating (approximately 15%). Considering all coating formulations, the water vapor permeability reduced by 10 to 60% compared to uncoated paperboard, except for two layers coated with 0:100. The tensile index (independent of AA:CHI) was higher in the machine direction (22.59 to 24.99 MPa) than in the cross-section (11.87–13.01 MPa). Paperboard coated only with chitosan showed superior properties compared to the other formulation coatings evaluated.

## 1. Introduction

Food packaging protects during distribution and storage. Packaging material selection is important to maintain food quality and safety, avoiding unfavorable conditions, such as spoilage, microorganism contamination, chemical contaminant presence, or the influence of external factors such as oxygen, humidity, and light [1,2]. Food packaging using a synthetic material, mainly petroleum products, is common, causing a negative environmental impact. It contributes to air, water, and soil pollution, as it is not a biodegradable polymer [3]. Environmental problems and recycling difficulties have increased recently, and alternatives are necessary, especially to partially replace synthetic polymers [4].

Paper has been widely used as packaging material for different products such as food, medicine, and clothing, among others. A wide variety of papers can be used to produce food packaging, including recycled paper [5]. However, reduced barrier properties (moisture, gases, and lipids), due to high porosity, limit their applications as packaging [6,7].

One of the alternatives to improve the properties of paper/paperboard-based packaging by improving barrier properties involves coating with plastics, glass, and/or metals [7,8,9]. Some materials significantly increase the barrier properties but compromise the recyclability of coated papers or increase the overall recycling costs [10]. Natural biodegradable polymer coatings are being used as alternatives to improve the properties (barrier properties to moisture, fat, and gases) of papers, in addition to presenting desired properties such as biodegradability, non-toxicity, and biocompatibility. Among the natural polymers, chitosan has been used due to its high gel formation capacity [11]. Chitosan is renewable, antibacterial, biodegradable, and non-toxic [12]. In addition, chitosan can easily interact with other materials, being indicated to produce active packaging material [13]. Soares et al. [14] developed a sustainable active packaging with ethylene adsorption capacity based on chitosan film containing NaY-Ag zeolite-coating Kraft paper. They reported that the coated Kraft paper reduced the water absorption capacity, although no significant differences in mechanical properties were observed compared to the control (without coating). 

The improvement of the water vapor barrier properties of a copy paper coating based on chitosan–beeswax was observed compared to the other coatings using beeswax and different natural polymers, such as sodium alginate, hydroxymethyl cellulose, hydroxyl starch, and zein [15]. A chitosan and wax from banana leaves coating were applied on the cellulose paper surface, verifying that chitosan efficiently dispersed wax on the surface of the paper, promoting effects on water absorption, oil absorption, and mechanical properties, among other properties [16]. 

Different coating techniques were reported in the literature to form paper coated with a chitosan solution. Most studies used a coating table [17], a size press machine [18], a blade [19], a K control coater [20], a manual glass-rod-coating technique [21], and a manual coating rod [16], among others. In general, the methods are still applied on a laboratory scale. However, in industrial processes, tape casting (spread casting or knife-coating) is well-known [22].

Furthermore, agar–agar is a natural polymer with several advantages, such as chemically inert and non-toxic hydrophilic polysaccharides extracted from seaweed [23]. Kumar et al. [24] verified that agar–agar formed a transparent and flexible film.

Paperboard and corrugated paperboard have a porous microstructure, facilitating the diffusion of liquids and oils. One way to make these materials resistant is to fill the pores to avoid the permeation of liquids [25]. Renewable materials, such as chitosan and alginate, coated different types of paperboards produced from primary and secondary cellulosic fibers, improving the grease resistance and reducing water vapor transmission [26]. Two types of carboxymethyl-chitosan-based (sodium alginate and carboxymethyl cellulose) biodegradable coated papers increased the air resistance, significantly affecting the grease resistance and improving the mechanical properties. The authors developed eco-friendly multifunctional packaging, providing quality and food safety [27].

This work aimed to develop an alternative sustainable packaging material based on a coated paperboard system based on agar–agar, chitosan, and agar–agar/chitosan blends with different numbers of layers, using the tape casting technique. To achieve our objectives, we analyzed the water absorption capacity, mechanical properties, and water vapor permeability of the coated paperboard in comparison to the uncoated paperboard.

## 2. Materials and Methods

### 2.1. Materials

Film form solutions were prepared using agar–agar purchased from TOWA bomboniere (São Paulo, Brazil) and chitosan acquired from Polymar (degree of deacetylation = 89%, Fortaleza, Brazil).

The paperboard used (duplex paperboard, 250 g/m^2^) was purchased from Ramenzoni (Cordeirópolis, Brazil). The other reagents were purchased from different companies, as listed: glacial acetic acid (LabSynth, Diadema, Brazil), isopropyl alcohol (Dinâmica, Indaiatuba, Brazil), magnesium nitrate (Dinâmica, Indaiatuba, Brazil), toluene (Dinâmica, Indaiatuba, Brazil), Brazil), n-heptane (Dinâmica, Indaiatuba, Brazil), castor oil (Dinâmica, Indaiatuba, Brazil), ethanol (Synth, São Paulo, Brazil), and erythrosine powder dye (Arcolor, São Paulo, Brazil).

### 2.2. Production of Coated Papers

Paperboard–natural polymer systems coated with agar–agar (AA), chitosan (CHI), and agar–agar/chitosan (AA:CHI) blends were prepared. For the AA film-forming solutions (AA-FFS), a concentration of 1 g of AA/100 g of FFS was used. The AA was dispersed in distilled water at 90 °C (heating plate IKA C-MAG HS7, Staufen, Germany) under mechanical agitation (IKA-RW20 DIGITAL, Staufen, Germany) for 2 min (complete solubilization). The AA-based FFSs were kept at room temperature for cooling to 35 °C; then, the FFS was used to coat the papers.

For the chitosan film-forming solutions (CHI-FFS), 2 g CHI/100 g of FFS was dispersed in an aqueous glacial acetic acid solution (0.62 mL). The concentration of acetic acid used was fixed according to Yoshida et al. [28] considering the chitosan degree of deacetylation and mass (maintenance of the stoichiometric ratio, aiming at the protonation of all NH_2_ sites). The dispersion was maintained under magnetic stirring (IKA, Topolino, Germany) for 1 h at room temperature (25 ± 2 °C). The CHI-FFS was applied to the paperboard surface at 25 °C.

The FFSs based on AA and CHI were prepared as described above for the AA-CHI blends. Chitosan and agar–agar solutions were produced and mixed in equal proportions (50% chitosan solution and 50% agar–agar solution), and the solution was kept under magnetic stirring for 10 min to mix the solutions completely.

The film form solutions (FFSs) were spread on a paperboard surface at 25 °C using an automatic spreader (Zehntner^®^, Sissach, Switzerland). The thickness was kept constant using an automatic film spreader (Zehntner ZAA2300, Sissach, Switzerland) and a high-precision adjustable applicator (TKB Erichsen, APF 01/20) at 500 µm. The spreading speed was kept constant at 20 mm/s (time of spreading on the surface of the paper = 15 s). After applying the solutions, the coated paperboard was dried in a forced circulation oven (Marconi, MA035) at 85 °C (4 min) for paperboard coated with agar–agar and AA-CHI blend. The drying temperature for paperboard coated with chitosan was 130 °C (2 min). After the first layer dried, the papers with one layer of coating were kept for 10 min at room temperature (25 ± 3 °C), and then the second layer of coating was applied.

The coated and uncoated paperboard samples were pre-conditioned in accordance with ASTM D685-93 in desiccators containing a saturated saline solution of magnesium nitrate (relative humidity of 50 ± 2%, temperature of 25 ± 2 °C) [29]. The samples were placed in silica for 10 days before analysis for scanning electron microscopy analysis.

### 2.3. Coated Paperboard Characterization 

#### 2.3.1. Thickness

The thickness average as the arithmetic mean of 10 random measurements in the sample area was determined using a digital Mitutoyo Absolute dial indicator (Mitutoyo, Kawasaki, Japan).

#### 2.3.2. Visual Aspects

The coated paperboard was visually evaluated for the absence of insoluble particles. Additionally, the heterogeneity of coated samples after the drying process was evaluated, verifying the presence of humid regions that could affect the final properties. Paper deformation was also evaluated.

#### 2.3.3. Coating Homogeneity

The coating homogeneity analysis was carried out according to the colorimetric methodology described by Marcy [30] using a 0.5% erythrosine solution (in isopropanol). Erythrosine solution was applied on the coated surface of the paperboard (samples of 15 × 20 cm). Then, the samples were dried at 50 °C for 1 min in an oven (Marconi, MA 035/100, Piracicaba, Brazil). The pink spots visualized on the opposite side of the colorimetric solution application indicated heterogeneity in the coating.

#### 2.3.4. Scanning Electron Microscopy

Analyzes were performed using a TM-3000 scanning electron microscope (Hitachi, Tokyo, Japan). Paperboard samples (with and without coating) were cut (10 × 10 mm) and stored in silica gel for 10 days before analysis. Surface and sectional area structure analyses were performed using 5 kV and 15 kV electron beams, respectively.

#### 2.3.5. Color

The color parameters, lightness (L*), a* (chroma a*), and b* (chroma b*) were determined using an AEROS non-contact colorimeter (HunterLab, Reston, USA). The color parameters of the control film (uncoated) and coated (coated side) were determined. Samples of 7 cm in diameter were used for the determinations. Color measurements were performed automatically (using the equipment’s software). The equipment performed a reading (during 10 s) on the area of the paper, and the average value of these readings corresponded to the determined value. The total color difference (ΔE*) of the coated papers in relation to the control (0:0, AA:CHI) was calculated from Equation (1), according to ASTM D2244 [31].

(1) $$\Delta E*\;=\;\sqrt{\left({\left({L*}_{B}-{L*}_{S}\right)}^{2}+{\left({a*}_{B}-{a*}_{S}\right)}^{2}+{\left({b*}_{B}-{b*}_{S}\right)}^{2}\right)}$$

where L*_B_, a*_B,_ and b*_B_ refer to the coated paperboard, and L*_S_, a*_S,_ and b*_S_ refer to the standard (control).

#### 2.3.6. Grammage

The uncoated (control) and coated paperboard grammage were determined according to the ASTM D646-96 methodology [32]. The samples (12.5 × 12.5 cm) were weighted using an analytical balance (AUY220, Shimadzu, Kyoto, Japan), and the grammage was calculated according to Equation (2).

(2) $$G=\frac{M}{A}$$

where G = grammage (g/m^2^); M = mass of the paper (g); A = paperboard area (m^2^).

#### 2.3.7. Water Vapor Permeability (WVP)

The water vapor permeability of the papers was determined according to the ASTM E 96/E 96M-16 methodology [33]. Samples (diameters of 7 cm) of uncoated (control) and coated paperboard were fixed in cells containing silica gel. Cells were stored in desiccators containing saturated saline magnesium nitrate solution (50 ± 5% relative humidity). The desiccators were maintained at 25.0 ± 0.2 °C (BOD Marconi-MA 415 incubator, Piracicaba, Brazil) for 72 h. Permeability was determined according to Equation (3).

(3) $$\mathrm{WVP}=\frac{\mathrm{Ge}}{tA_{e}P_{0}(R1-R2)}$$

where G/t = mass gain of the system as a function of time (72 h), e = sample average thickness (mm); A = exposed area of the system (0.003117 m^2^); P_0_ = water vapor pressure at 25 °C (3.1590 kPa); R1 − R2 = relative humidity gradient (50%). 

#### 2.3.8. Mechanical Properties

The mechanical properties (tensile index and elongation at break) were determined according to the ASTM D828-16e1 methodology [34]. Coated and uncoated paperboard samples were cut (15 × 180 mm) in the transversal or cross direction (CD) and in the longitudinal or machine direction (MD) of the cellulose fiber of the paperboard sheet. The analyses were performed using a universal testing machine Landmark Servo Hydraulic Test System (MTS, Eden Praire, MN, USA), with a 1 kN load cell, test speed of 20 mm/min, and initial distance of 120 mm.

#### 2.3.9. Water Absorption Capacity (WAC_Cobb_)

The water absorption capacity (WAC_Cobb_) was evaluated according to the ASTM D3285-93 methodology [35]. Paperboard samples (0.125 × 0.125 m) were fixed in Cobb equipment (Regmed, Osasco, Brazil). Water (100 mL) was deposited on the samples (outlined by the equipment ring). After 120 s, the water was removed, and the samples were deposited on two sheets of absorbent paper and quickly pressed with a cylindrical roller to remove the excess. After this period, the samples were weighed using a semi-analytical balance (ARD110, Ohaus Corporation, Morris, NJ, USA). WAC_Cobb_ was determined according to Equation (4). The samples were weighted using a semi-analytical balance (ARD110, Ohaus Corporation, Morris, NJ, USA).

WAC_Cobb_ = (M_f_ + M_i_) × 100 (4)

where M_f_ = final weight (g); M_i_ = initial weight (g).

#### 2.3.10. Contact Angle

The contact angle (CA) on the uncoated (control) and coated papers was evaluated using ultrapure water. Analyses were performed using an Attension Theta Lite tensiometer (KSV Instruments, Biolin Scientific AB, Gothenburg, Sweden). Samples (2 × 3 cm) were fixed in the equipment, and a drop (5 µL) of ultrapure water was deposited on the surface of the control paper (brown side) and the coated paperboard. The variation in the contact angle was evaluated in the interval of 300 s (25 ± 2 °C).

#### 2.3.11. Grease Resistance

Grease resistance was performed according to the methodology TAPPI pm-96.27 [36]. A kit (12 solutions) with different concentrations of castor oil, toluene, and n-heptane was used for the analysis. A drop of solution was deposited on the paperboard (with and without coating), and, after 15 s, the excess solution was removed. The opposite side of the solution application of the paperboard was evaluated. The grease resistance was the number of the kit solution that did not cause stains on the paper’s back.

### 2.4. Statistical Analysis

Statistical analysis, the difference between means, was performed using the SAS Version 9.4 computer program (SAS Inc., Cary, NC, USA) and Duncan’s test (with a 95% confidence limit). The coatings were prepared three times (three different productions of the same formulation), and analyses were performed in triplicate (totaling 9 samples) for each repetition. For the mechanical properties, in exception, 30 samples were analyzed.

## 3. Results and Discussion

### 3.1. Visual Aspect and Coating Homogeneity 

Visually, no differences were verified between the coatings applied on the surface of the papers. No insoluble particles were observed on the surface of the coated paperboard in all coated formulations used (Table 1). 

The coated paperboards were homogeneous after the drying process, and there was no deformation of the papers after applying the coatings (Table 1). The coated homogeneity was verified by applying different AA:CHI ratios and different numbers of layers (there were no pink stains on the opposite paperboard surface). The solid content of the coating applied on a paperboard surface could fill the cellulose–fibrous structure [37]. The results suggested that the coating formulations may have penetrated the paper cellulose fibers, which may have contributed to improving the barrier properties since the homogeneity coating was verified. The results observed in relation to homogeneity were superior to those observed by Reis et al. [38] for kraft papers coated with chitosan solutions (different concentrations, 3 and 4%, *w*/*w*), possibly due to the coating method applied.

The literature reported that coating papers using chitosan-based solutions presented homogeneity related to filling paper fibers [37]. 

### 3.2. Scanning Electron Microscopy

**Figure 1:**
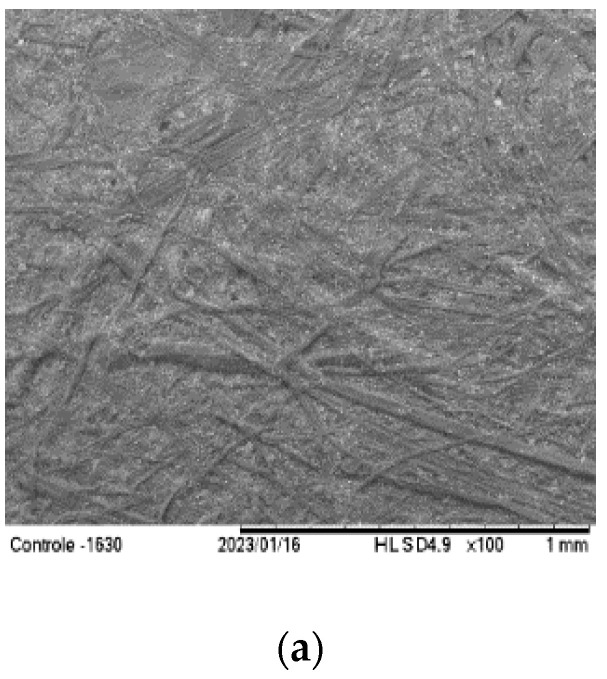

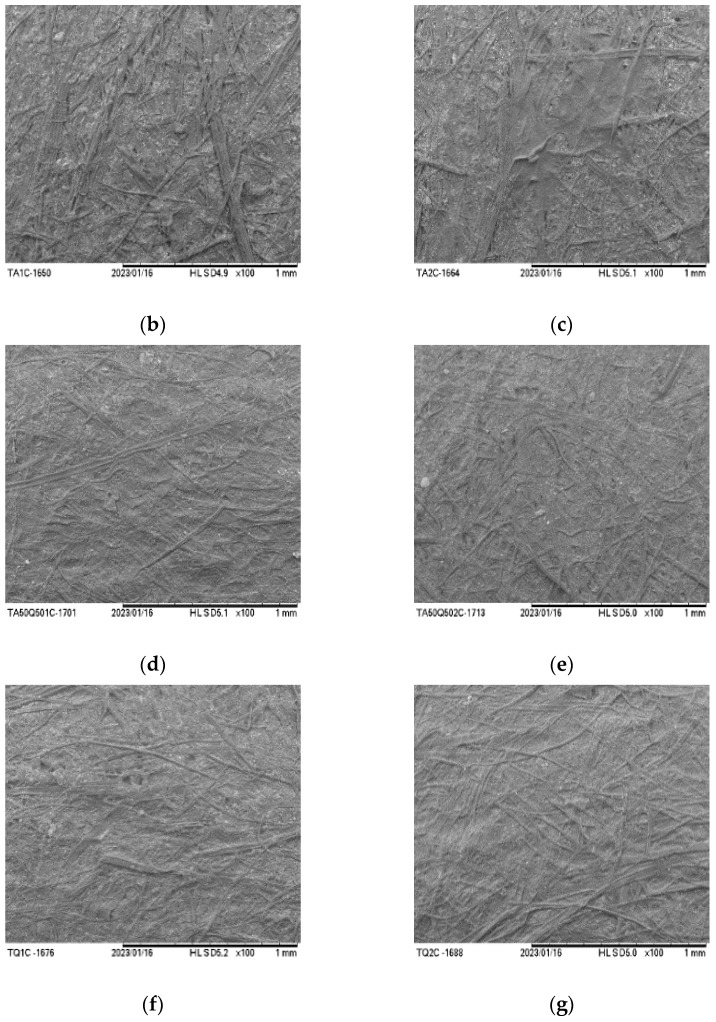
Surface micrographs of coated paperboard with different agar–agar/chitosan ratios (AA:CHI) and different numbers of coating layers (NCL). Where (**a**) 0:0 (Control); (**b**) 100:0 AA:CHI and 1 NCL; (**c**) 100:0 AA:CHI and 2 NCL; (**d**) 50:50 AA:CHI and 1 NCL; (**e**) 50:50 AA:CHI and 2 NCL; (**f**) 0:100 AA:CHI and 1 NCL; and (**g**) 0:100 AA:CHI and 2 NCL.

The interlaced cellulose fibers were verified (Figure 1) on the surface of the uncoated paper (control). Partial coverage of the cellulose fibers was observed for the first coating layer for all coating formulations used, which could have been associated with the absorption of film formation, filling the paperboard pores. Applying the second layer of the coating solution increased the filling of the porous structure of the paper, making the cellulose fiber visualization less evident. The results were related to the coated characteristics of the polymers and the thicknesses used. 

According to Zhang et al. [15], the paper structure was mainly composed of a three-dimensional cellulose fiber matrix with a porous surface. In the coating process, the copy paper was coated with chitosan solution (1.0, 2.0, 3.0 wt%), the small voids were filled, and roughness was reduced. Adding the beeswax layer, the two layers were indistinguishable. The filling of the pores in the paper matrix could also be attributed to the interaction between the chitosan’s positive charges and the cellulose fibers’ negative charges [39].

The effect of the number of layers of chitosan-based coatings (1% *w*/*w*) on food-grade paper was analyzed. Applying one and two layers caused the filling of the pores, verified using scanning electron microscopy. The formation of a film on the surface was observed after applying three and five layers [20]. In the present work, the chitosan concentration was 2% (*w*/*w*). Therefore, the viscosity of the solution was higher, and it was operationally complicated to apply more coating layers.

### 3.3. Color

**Figure 2:**
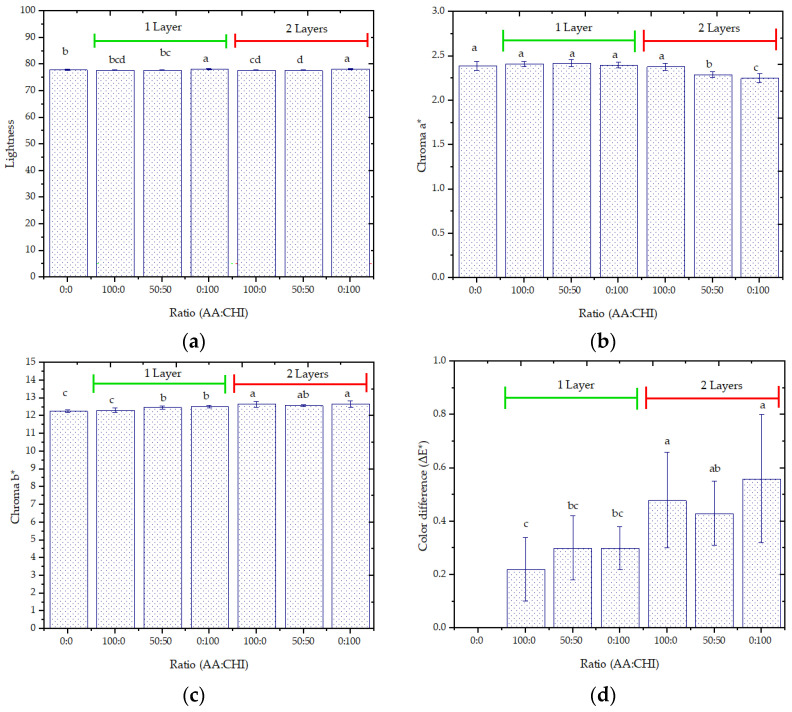
(**a**) Lightness (L*), (**b**) chroma a*, (**c**) chroma b*, and (**d**) total color difference (ΔE*) of coated paperboard with different agar–agar/chitosan ratios (AA:CHI), and different numbers of coating layers. Note: Different lowercase letters in the same column indicate a significant difference (*p* < 0.05).

The parameters lightness, chroma a*, chroma b* (Figure 2a–c) of two layers of the blend-coated paperboard and coated paperboard only with chitosan significantly differed from the values observed for the uncoated paper. The ∆E* values were reduced (less than 0.56) (Figure 2d), indicating distinct color according to the classification of Adekunte et al. [40]. These results could have been related to the absorption of the film-formed solution, filling the pores between the cellulose fiber paper, not occurring for the formation of a continuous film on the surface of the coated paperboard. There were insufficient layers to form a continuous matrix on the surface of the paperboard, as indicated by SEM analysis.

Tanpichai et al. [20] observed a reduction in L* and a*, and an increase in b*, increasing the number of coating layers (four layers) in the food-grade paper. 

In chitosan- and banana-leaf-extracted wax coated paper with two layers, the values of L* and a* were reduced (92.24 ± 0.01 and −0.54 ± 0.01, respectively), and the a* values increased (2.24 ± 0.04) as compared to uncoated paper (L* = 92.51 ± 0.13, chroma a = −0.33 ± 0.02 and chroma b* = 1.48 ± 0.05), attributed to the chitosan’s incorporation [16]. 

### 3.4. Thickness and Grammage

With the exception of the papers covered with 100:00 (AA:CHI), the coating with one layer (50:50 and 0:100 AA:CHI) did not cause a significant change in the thickness of the papers in relation to the control (Table 2). Possibly, the results were related to the absorption of the filmogenic solution by the fibers of the papers. However, the application of the second layer caused a significant increase in thickness compared to the control. In general, similar results were observed in relation to grammage. The variations may have been related to the non-uniform absorption of film-forming solutions in the cellulosic matrix, which may have explained the results associated with thickness and grammage.

Thickness and grammage increases were observed in coated papers using natural polymers in most works found in the literature. Tanpichai et al. [20] produced papers covered with different layers of chitosan (one to five layers), observing an increase in the thickness and weight of the coated paper, regardless of the number of layers. The thicknesses and weights of the uncoated paper were 74.5 μm and 44.65 g/m^2^, respectively; after applying a one-layer coating, the thickness increased by ~28%, and the weight increased by ~5%. Reacetylated-chitosan-coated paperboard (degree of acetylation of 48%) provided a higher thickness average (values between 3.78 and 4.08 μm) and a higher grammage (values between 252.23 and 259.93 g/m^2^) than uncoated paperboard [37]. 

### 3.5. Water Vapor Permeability (WVP)

One-layer (NCL = 1) coated paperboard promoted a reduction in WVP in all coating formulations (Table 3) compared to uncoated paperboard (control). The water vapor barrier was improved by adding more coating layers due to the filling saturation of the cellulose fibers network pores, thus forming a continuous film on the paper’s surface. Applying two layers of coating of the 50:50 and 0:100 (AA:CHI) formulations, no significant differences were observed in the WVP compared to the uncoated paper. It was verified for papers coated with 100:00 that there was a reduction in WVP despite the high hydrophilicity of the agar–agar (Table 3). The results may have been related to the distribution of the polymer in the paper fibers, considering that the filmogenic solution had a higher viscosity than the other solutions, which could lead to changes in permeation inside the paper fibers. The high error values also indicated sample heterogeneity, suggesting that the distribution of polymers within the matrix may have been heterogeneous, although coating homogeneity was observed (Table 1).

The SEM results indicated that the film-forming solution penetrated the cellulosic matrix. The application of the first layer could have been associated with the pores filling, and the application of two layers could cause changes in the distribution of polymers inside the pores of the paperboard. A reduction in the permeability of papers coated only with chitosan was observed by applying five layers [18].

### 3.6. Water Absorption Capacity (WAC_Cobb_)

The water absorption capacity of coated paperboard with AA (100:0), and AA:CHI (50:50) did not present significant differences (Table 3). The WAC_Cobb_ significantly reduced in the chitosan-coated paperboard. The agar–agar had a high hydrophilic character, and, for films based on chitosan and agar, higher swelling values were observed due to the agar–agar water solubility characteristics, the higher number of hydrophilic groups (-OH), and high amorphous contents [41].

Several parameters affect the water absorption capacity, such as the type of paper, polymers, number of coating layers, and coating thickness. The lower values of water absorption of papers coated with chitosan and lemongrass essential oil, compared to the results found in the present study, were associated with the lipid compound incorporation [42]. 

### 3.7. Grease Resistance (GR)

The grease resistance increased in coated paperboard using different AA:CHI ratios, regardless of the number of layers applied (Table 3) compared to the control paper (uncoated). The results are possibly related to the absorption of the film-forming solution in the cellulosic matrix, increasing the grease barrier. It was also verified (Table 3) that increasing the coating layers provided a higher grease resistance. It was associated with the number of coating layers that increased the cellulosic matrix pore filling, forming a physical barrier. A similar result was verified in the grease resistance of paperboard coated with chitosan and lemongrass essential oil related to the number of coating layers [42].

The GR for papers coated with natural polymers is reported in the literature. Papers coated with polyvinyl alcohol, alkyl ketene dimer, and nanoclay (different formulations) showed excellent grease resistance, i.e., kit 12 [43].

### 3.8. Contact Angle 

**Figure 3:**
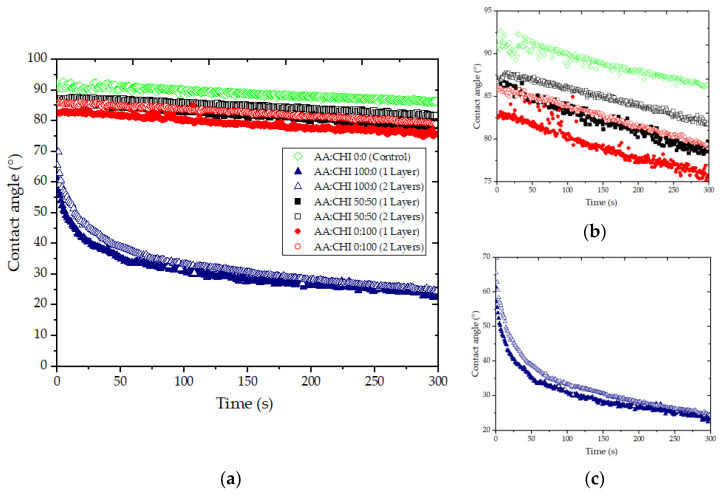
Variation in the contact angle as a function of time for paperboard coated with different agar–agar/chitosan ratios (AA:CHI) and different numbers of coating layers: (**a**) different ratios AA:CHI and different numbers of layers (one and two layers), (**b**) papers coated with 50:50 and 00:100, different scales, and (**c**) papers coated with 100:00, different scales.

The polymeric coating on the paperboard surface significantly reduced the contact angle compared to the uncoated paper (Figure 3). The hydrophilicity of the coated surface increased due to the hydrophilic character of the natural polymers (agar–agar and chitosan). A reduction in the contact angle was observed, increasing the AA concentration in AA:CHI-based films associated with greater hydrophilicity. Most natural polymers have hydrophilic characteristics, which is the main challenge in using them [41].

Opposite results were observed for papers coated with alginate, carrageenan, and carboxymethyl cellulose, which increased the contact angle (with an increase in thickness), indicating a greater hydrophobicity surface [44].

The contact angle of coated paperboard was reduced in all coating formulations applied, indicating greater hydrophilicity. Similar behavior was observed by Rhim et al. [45] for papers coated with different polymers (soy protein isolate and sodium alginate).

On the other hand, a linear decrease in the contact angle as a function of time was verified (Figure 3, Table 4). Aluminum foil was used as a reference due to its characteristic of not absorbing water. The variation in the contact angle was insignificant in the function of time, which indicated that the evaporation phenomena could be disregarded. The results indicated that the decreasing contact angle (Table 4) may have been related to the scattering absorption phenomena. Paperboard coated only with chitosan showed lower K. Several phenomena could occur on the surface of different films based on natural polymers, such as evaporation, absorption, or even scattering [46].

### 3.9. Mechanical Properties

**Figure 4:**
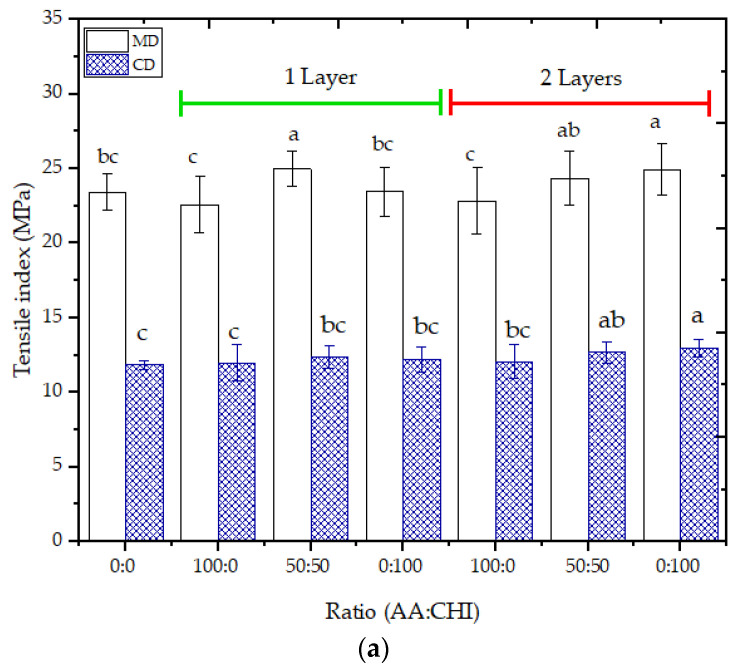

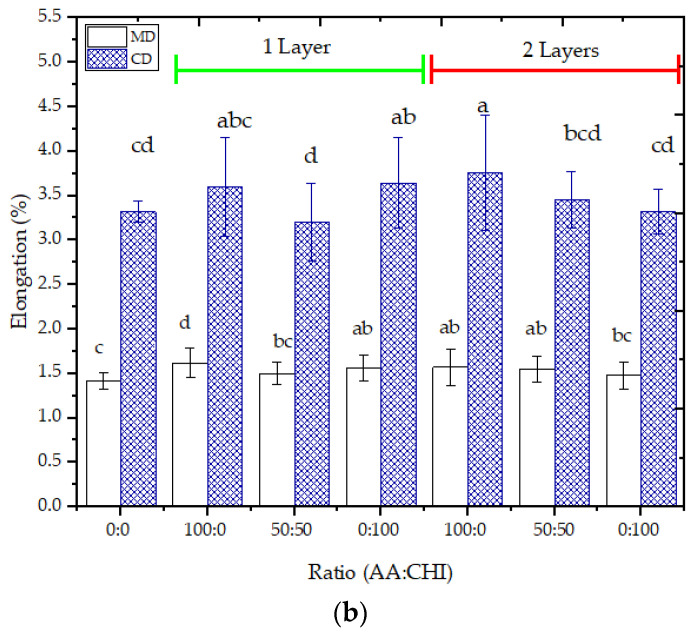
(**a**) Tensile index and (**b**) elongation at break in the longitudinal direction or machine direction (MD) and the transversal or cross direction (CD) of paperboard coated with different agar–agar/chitosan ratios (AA:CHI) and different numbers of coating layers. Note: Different lowercase letters in the same column indicate a significant difference (*p* < 0.05).

The tensile index (T) and elongation (E) at break did not present a significant difference in the longitudinal (MD) and transversal (CD) direction of the cellulose fiber of the paperboard sheet compared to the uncoated paperboard (Figure 4). No correlation was observed between the T (Figure 4a) and E (Figure 4b) of coated paperboard samples, the coating formulation, and the number of coating layers applied. The mechanical properties of coated paperboard followed the cellulose matrix of paperboard properties. This could have been associated with the absorption of film-forming solutions in the paperboard pores; consequently, a continuous film was not formed on the surface of the paperboard. The tensile index at the break for all coated formulations was higher for the longitudinal direction (paper fibers direction), possibly related to the reinforcement of the fibers resulting from the impregnation with the polymers. 

The same behavior was observed by Fernandes et al. [18] in chitosan coating papers using a size press machine, presenting T values in the range of 100.3 ±  1.2 to 117.04 ±  0.8 N.m/g (one to five layers) in the machine direction and 26.0 ± 0.4 to 37.5 ± 0.4 N.m/g (one to five layers) in the cross-machine direction. Opposite results were verified in relation to elongation at break. The effects of the direction of the cellulose fibers in chitosan-coated kraft paper were contrary to those presented in the present work. However, the authors used higher chitosan concentrations, which caused the formation of a more homogeneous surface and cellulose fiber coating [38].

The tensile index did not present significant differences comparing chitosan solution (one to five layers) coated paper with uncoated paper (control). The application of one layer (T = 40.78 ± 8.10 MPa) did not observe significant differences in the tensile index in relation to the control (T = 37.94 ± 2.95 MPa), indicating this behavior to the presence of voids without chitosan filling in the paper. However, applying the second layer showed an increase in tensile index (43.90 ± 4.23 MPa), attributing this effect to the chitosan filling of void spaces between cellulose fibers. In the third layer applied, no significant differences were observed due to the formation of chitosan films on the paper’s surface [20]. The elongation at break values did not differ after applying the chitosan coating on different types of papers (ahlstrom paper and stora enso paper), remaining in the range of 2.8–3.0% [17].

The tensile strength values of the coated papers with chitosan solution using a 100 µm blade were higher than the control (without coating), 60.1 ± 3.6 and 39.0 ± 3.4 MPa, respectively. However, no differences were observed in the elongation values, comparing the paper with and without chitosan coating (3.0 ± 0.2 and 2.8 ± 0.2%, respectively) [19]. 

## 4. Conclusions

The coatings based on different AA:CHI ratios, regardless of the number of layers, showed homogeneity and improved the grease resistance in coated paperboard samples. The coated paperboard presented superior hygroscopicity in relation to the uncoated paper due to the hydrophilic chitosan and agar–agar polymer characteristics used. Applying two layers of coatings containing chitosan in their formulations (50:50 and 0:100) provided a water vapor permeability reduction and increased grease resistance. Thus, the type of polymers and the number of coating layers are important parameters that affect the final properties of the coated paperboard. Considering the variation in the contact angle as a function of time, the paperboard coated with 100:0 and 50:50 showed a faster decrease in the contact angle than the coating based on 0:100. In general, all formulation coatings increased the hydrophilic character of the material. Paperboard coated only with chitosan presented the best barrier properties (grease and water vapor).

## Figures and Tables

**Table 1:** The visual aspects, homogeneities, and microstructures (SEM images) of uncoated (0:0, control) and coated paperboards with different numbers of coating layers (NCL). The polymers were used in different agar–agar/chitosan (AA:CHI) ratios.

Ratio (AA:CHI)	NCL	Visual Aspect	Homogeneity	
			Control/Coating Side	Uncoated Side
0:0 (Control)	-	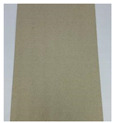	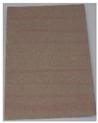	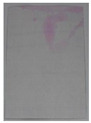
100:0	1	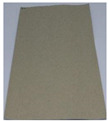	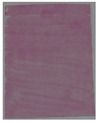	* 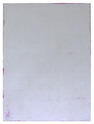 *
	2	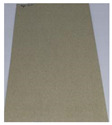	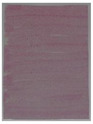	* 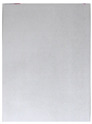 *
50:50	1	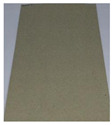	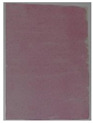	* 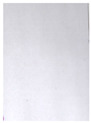 *
	2	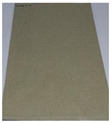	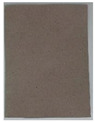	* 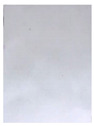 *
0:100	1	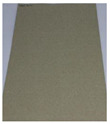	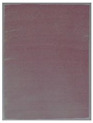	* 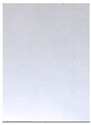 *
	2	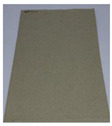	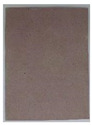	* 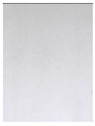 *

**Table 2:** Thickness and grammage of coated paperboard with different agar–agar/chitosan ratios (AA:CHI) and different numbers of coating layers (NCL).

Ratio (AA:CHI)	NCL	Thickness (μm)	Grammage (g/m^2^)
0:0 (Control)	-	427 ± 18 ^d^	265 ± 4 ^cd^
100:0	1	457 ± 25 ^c^	269 ± 3 ^a^
	2	476 ± 84 ^b^	268 ± 2 ^abc^
50:50	1	439 ± 21 ^d^	266 ± 2 ^bcd^
	2	497 ± 75 ^a^	270 ± 5 ^a^
0:100	1	435 ± 18 ^d^	264 ± 2 ^d^
	2	493 ± 79 ^a^	268 ± 2 ^ab^

Note: Different lowercase letters in the same column indicate significant difference (*p* < 0.05).

**Table 3:** Water vapor permeability (WVP), water absorption capacity (WAC_Cobb_), and grease resistance (GR) of paperboard coated with different agar–agar/chitosan ratios (AA:CHI) and different numbers of coating layers (NCL).

Ratio (AA:CHI)	NCL	WVP (g.mm/.h.m^2^.kPa)	WAC_Cobb_ (g/m^2^)	GR (Kit Number)
0:0 (Control)	-	0.30 ± 0.03 ^a^	43.00 ± 1.73 ^ab^	3
100:0	1	0.16 ± 0.02 ^b^	46.22 ± 4.89 ^a^	5
	2	0.13 ± 0.05 ^b^	44.44 ± 4.03 ^ab^	6
50:50	1	0.12 ± 0.05 ^b^	42.33 ± 2.50 ^ab^	8
	2	0.27 ± 0.17 ^a^	40.22 ± 5.09 ^bc^	10
0:100	1	0.16 ± 0.12 ^b^	36.67 ± 3.97 ^c^	8
	2	0.35 ± 0.04 ^a^	37.33 ± 5.74 ^c^	11

Note: Different lowercase letters in the same column indicate significant difference (*p* < 0.05).

**Table 4:** Initial contact angle (θ_0_), contact angle decreasing rate (K), and correlation coefficient (R^2^) of paperboard coated with different agar–agar/chitosan ratios (AA:CHI) and different numbers of coating layers (NCL).

AA:CHI	NCL	$\theta_{0}$	K (°s^−1^)	R^2^
00:00	-	91.983	0.0182 ± 0.0003	0.9167
100:00	1	63.072	0.0667 ± 0.0022	0.7580
	2	68.520	0.0802 ± 0.0026	0.7579
50:50	1	88.966	0.0265 ± 0.0002	0.9678
	2	89.485	0.0196 ± 0.0001	0.7843
00:100	1	85.536	0.0256 ± 0.0004	0.9217
	2	85.148	0.0227 ± 0.0001	0.9895

## Data Availability

Not applicable.
